# Integrated LTE and Millimeter-Wave 5G MIMO Antenna System for 4G/5G Wireless Terminals

**DOI:** 10.3390/s20143926

**Published:** 2020-07-15

**Authors:** Syeda Iffat Naqvi, Niamat Hussain, Amjad Iqbal, MuhibUr Rahman, Masoud Forsat, Seyed Sajad Mirjavadi, Yasar Amin

**Affiliations:** 1 ACTSENA Research Group, Department of Telecommunication Engineering, University of Engineering and Technology, Taxila, Punjab 47050, Pakistan; iffat.naqvi@uettaxila.edu.pk (S.I.N.); yasar.amin@uettaxila.edu.pk (Y.A.); 2 Department of Computer and Communication Engineering, Chungbuk National University, Cheongju 28644, Korea; hussain@osp.chungbuk.ac.kr; 3 Centre for Wireless Technology, Faculty of Engineering, Multimedia University, Cyberjaya 63100, Malaysia; aiqbal@ieee.org; 4 Department of Electrical Engineering, CECOS University of IT and Emerging Sciences, Peshawar 25000, Pakistan; 5 Department of Electrical Engineering, Polytechnique Montreal, Montreal, QC H3T 1J4, Canada; 6 Department of Mechanical and Industrial Engineering, College of Engineering, Qatar University, P.O. Box 2713 Doha, Qatar; smir512@aucklanduni.ac.nz

**Keywords:** array, fourth-generation (4G), Long-Term Evolution (LTE), multiple-input-multiple-output (MIMO), millimeter-wave (mm-wave), fifth-generation (5G)

## Abstract

This work demonstrates an integrated multiple-input multiple-output (MIMO) antenna solution for Long Term Evolution (LTE) and Millimeter-Wave (mm-wave) 5G wireless communication services. The proposed structure is comprised of a two-element LTE MIMO antenna, and a four-element 5G MIMO configuration with rectangular and circular defects in the ground plane. For experimental validation, the proposed structure is fabricated on a Rogers RO4350B substrate with 0.76 mm thickness. The overall substrate dimensions are 75 mm × 110 mm. The proposed structure is capable of operating at 5.29–6.12 GHz (LTE 46 and 47 bands) and 26–29.5 GHz (5G mm-wave) frequency bands. Additionally, the measured peak gain of 5.13 and 9.53 dB is attained respectively for the microwave and mm-wave antennas. Furthermore, the analysis of the MIMO performance metrics demonstrates good characteristics, and excellent field correlation performance across the operating bands. Furthermore, the analysis of the Specific Absorption Rate (SAR) and Power Density (PD) at the lower frequency band (5.9 GHz) and PD only at mm-Wave frequency band (28 GHz) verifies that the proposed antenna system satisfies the international human safety standards. Therefore, the proposed integrated MIMO antenna configuration ascertains to be a potential contender for the forthcoming communication applications.

## 1. Introduction

In recent years, the notable growth of wireless devices has substantially increased efforts to develop advanced standards for communication networks [1,2]. In addition, for the rapid transmission of data wirelessly, it is necessary to have a wider bandwidth and a higher data rate. Several reported works demonstrate the efforts of researchers to satisfy users’ expectations of high-throughput for 4G and LTE (Long Term Evolution) communication applications [3,4,5,6,7]. Subsequently, this has promoted the development of next-generation 5G mobile and broadband wireless communication, featuring higher data rates and greater channel capacity compared to 4G/LTE [8,9]. With increasing demand for greater capacity and wider bandwidth, concerns about bandwidth inadequacy at the sub-6 GHz frequency spectrum have been raised. To realize the forthcoming requirements of high capacity and throughput for 5G communication, the spectrum at the millimeter-waves is envisioned to be utilized [10,11]. However, obstructions at mm-wave frequencies, like path loss attenuations and increased absorption in the atmosphere, are essential to be taken care of. The use of a single antenna makes this more challenging [12,13]. Therefore, 5G antennas with wider bandwidth and high gain are required, respectively for the concurrent functioning of various system services, and to reduce attenuations and absorption in the atmosphere at the mm-wave spectrum [14]. Lately, multiple-input multiple-output (MIMO) antenna solutions have been ascertained to be key enabling technology for 4G/LTE and futuristic 5G communication applications, allowing multi-antennas to operate simultaneously, and thus escalating the channel capacity, with the merits of multi-Gbps throughput and higher data rates. Therefore, MIMO architectures enhance communication reliability [15,16].

Recently, various works on 4G/LTE and 5G antenna design have been reported in the literature on rigid and conformal substrates [3,4,5,6,7,17,18,19,20,21]. It can be noticed that most of the proposed antenna solutions are either single-element or antenna arrays. Although a high gain is achieved with the use of antenna arrays, the capacity performance of the array antennas is observed to be similar to that of a single antenna. Therefore, in order to attain the high capacity and throughput requirements, deployment of MIMO antennas is necessary. MIMO antenna systems have been extensively investigated for 4G/LTE mobile communications [22,23,24,25], and, likewise, MIMO technology is also expected to be very promising for 5G wireless communications. Hence, researchers have presented many MIMO antenna solutions for 5G applications, at sub-6 GHz as well as at the mm-wave bands [26,27,28,29,30,31].

The 4G/LTE and 5G antenna solutions discussed above are either single-element antennas, antenna arrays, or MIMO antennas operating at the microwave or the mm-wave frequencies. However, integration of the 4G/LTE and the mm-wave 5G antennas will be an effective solution for forthcoming long and short-range communication applications. However, designing such antennas for handheld devices is very challenging due to increased coupling currents between adjacent antennas, which are closely packed due to size restrictions in these devices [32]. Some of the recent works have demonstrated integrated antenna solutions for handheld applications [33,34,35,36,37,38,39,40]. The work in [33] presents a hybrid 4G/5G MIMO antenna solution. The 4G module consists of a two-element MIMO antenna system covering the Global System for Mobile Communications (GSM), Universal Mobile Telecommunications System (UMTS), and LTE bands, whereas a 5G antenna system consists of an eight-element array covering the sub-6 GHz 5G band. Similarly, a 12-element MIMO array functional in the LTE and 5G bands for mobile applications has been presented [34]. The work in [35] presents a coexisting 4G and 5G antenna system suitable for smartphone devices. The peak gain obtained is 4 and 8 dBi, respectively, for 4G and 5G antennas. However, the proposed configuration is a multilayered structure with increased complexity. Another work presents an ultra-wideband (UWB) antenna array for mm-Wave 5G applications such as tablets, laptops, and mobile phones [36]. The proposed antenna structure covers the 23–33 GHz frequency band, with a peak gain of 4.6 dB. This beamsteerable antenna exhibits a directivity value of 13.5 dBi for the operating frequency. However, the antenna array covers only the mm-wave band, and is not an integrated design. Furthermore, an integrated design consisting of a printed-inverted-F MIMO antenna system for 4G applications and a connected array for 5G applications is proposed in [37]. In [38], an integrated conformal 4G LTE and 5G MIMO antenna system is demonstrated for smartphones. The metamaterial-based 4G two-element MIMO antenna system attained a gain of 2.15 dBi, whereas a gain of 9 dBi was achieved for the 5G MIMO antennas. Moreover, in [39], an integrated 4G and mm-wave 5G antenna array exhibiting good operational bandwidth and gain values is proposed. However, the reported structure is without MIMO, and therefore the capacity performance was not analyzed for the proposed configuration. Likewise, in [40], co-designed LTE and mm-wave antennas are proposed with a peak gain value of 7 dBi for the mm-wave band. This design introduces more complexity, as two different substrates (FR4 and Rogers RO4350B) are used. Most of the integrated antenna solutions reported in the literature and discussed above (except [34]) have not received SAR or PD analysis, which is an important performance metric to validate the antenna design as a safe solution, as per international human safety regulations.

Therefore, to overcome the limitations in existing designs and to deal with the aforementioned challenges, this work presents a two-element MIMO antenna structure for LTE-bands 46 and 47, and a 4-element MIMO configuration for mm-wave 5G frequency bands. Each 5G MIMO antenna consists of a two-element array with a parallel feed network. Defected Ground Structure (DGS) is incorporated to enhance the isolation, consequently improving the radiation characteristics of the demonstrated structure. The proposed geometry exhibits significant gain and bandwidth for the LTE and mm-wave 5G resonating frequency bands. As MIMO is the key enabler technology for futuristic communication systems, the proposed antenna system demonstrates good MIMO performance with better diversity characteristics and low channel capacity loss. In addition, SAR and PD analysis validates the proposed solution according to human safety standards. Hence, the proposed co-designed LTE and mm-wave 5G antenna system is a prospective candidate for impending communication applications.

## 2. Proposed Antenna Design

**Figure 1:**
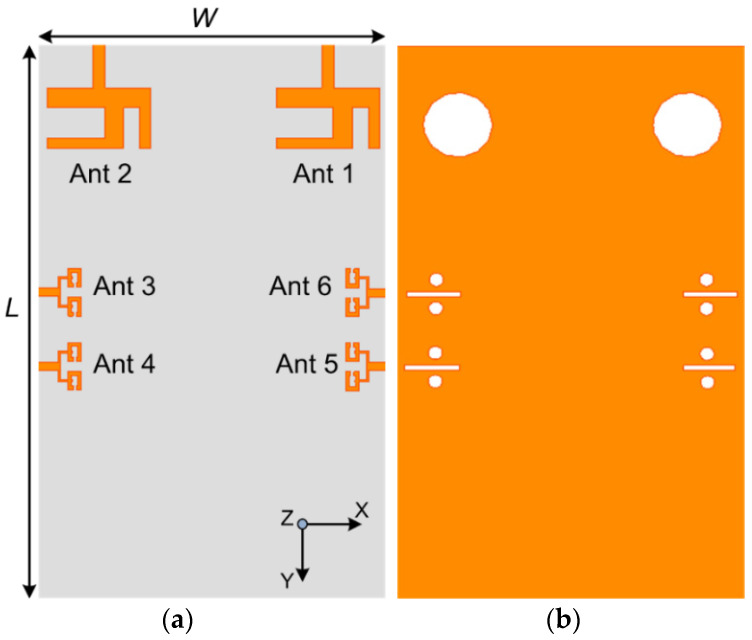
Geometry of the Integrated LTE (Long Term Evolution) and 5G MIMO (multiple-input multiple-output) Antenna System: (**a**) Top View; (**b**) Back View (Dimensions are in millimeter).

The proposed design was modeled, simulated and optimized using CST MICROWAVE STUDIO^®^. The design was modeled using a Rogers RO4350B substrate with *ε_r_* = 3.6, and a loss tangent δ = 0.0037. Figure 1 shows the layout of the final proposed design, with LTE and 5G MIMO antenna systems assimilated on the same substrate. The geometrical substrate size is 75 mm × 110 mm × 0.76 mm. The design is composed of a two-element MIMO structure placed on the top edge of the board, supporting LTE frequency bands, whereas the four-element MIMO antennas framed on the elongated edge of the board operate at the 5G mm-wave frequency bands. Each mm-wave MIMO antenna is a two-element linear array with a parallel feed network. Moreover, DGS structure is added at the ground layer as depicted in Figure 1b, to obtain better radiation performance. The optimized parametric values for the demonstrated MIMO antenna system are provided in Table 1. The design progression is unfolded by first describing the LTE and 5G single-element antennas, followed by the MIMO configuration, and, finally, the integration of two structures is presented.

### 2.1. LTE Antenna Configuration

**Figure 2:**
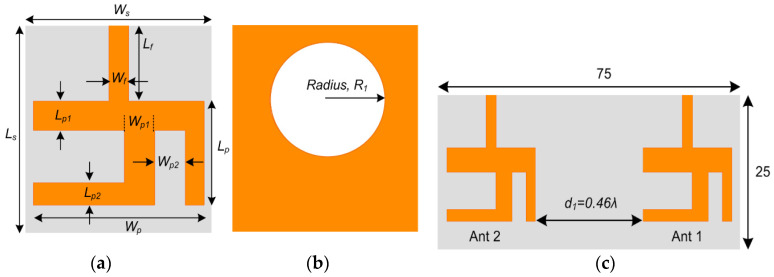
Geometry of LTE Antenna: (**a**) Single-Element, Front View; (**b**) Single-Element, Back View; (**c**) MIMO Configuration.

A systematic design of an inverted Y-shaped antenna provided with a 50 Ω matched microstrip feedline is suggested for the LTE frequency bands, as illustrated in Figure 2a. The Y-shaped antenna is modeled on top of the substrate, where the size of the substrate is *L_s_* × *W_s_*. For 50 Ω impedance matching, the width *W_f_* of the feedline is adjusted by considering the microstrip transmission line characteristic equations. Likewise, the primary structure of the rectangular patch antenna resonating at the desired frequency is obtained by using the following mathematical equations [41]:

(1) $$W_{p}=\frac{c}{2f_{c}\sqrt{\frac{\varepsilon_{r}+1}{2}}}$$

(2) $$L_{p}=\frac{c}{2f_{o}\sqrt{\varepsilon_{reff}}}-2\Delta L$$

where *W_p_* is the width, and *L_p_* is the length of the resonator. *ε_r_* is the relative permittivity, *f_o_* is the operating frequency, and Δ*L* is the effective length obtained by the following relation:

(3) $$\Delta L=0.421h\frac{\left(\varepsilon_{reff}+0.3\right)\left(\frac{W_{s}}{H}+0.264\right)}{\left(\varepsilon_{reff}-0.258\right)\left(\frac{W_{s}}{H}+0.8\right)}$$

where *ε_reff_* is given as:

(4) $$\varepsilon_{reff}=\frac{\varepsilon_{r}+1}{2}+\frac{\varepsilon_{r}-1}{2}(\frac{1}{\sqrt{1+12\left(\frac{H}{W_{s}}\right)}}$$

where, *ε_reff_* is the effective permittivity, and *W_s_* and *H* are the width and height of the substrate. Thus, an optimized Y-shaped antenna is obtained after the parametric analysis. Moreover, Figure 2b depicts that a circular slot of radius, *R*_1_, is loaded at the bottom layer as DGS to vary the current density at the ground, which leads to improved radiation performance. The dimensions of the optimized antenna are tabulated in Table 1. Afterwards, the single-element antenna is proceeded to a two-element MIMO configuration with substrate dimensions of 75 mm × 25 mm × 0.76 mm, as shown in Figure 2c. The two MIMO elements Ant1 and Ant2 are separated by a distance *d*_1_ approximately equal to 0.46λ at 5.9 GHz.

**Figure 3:**
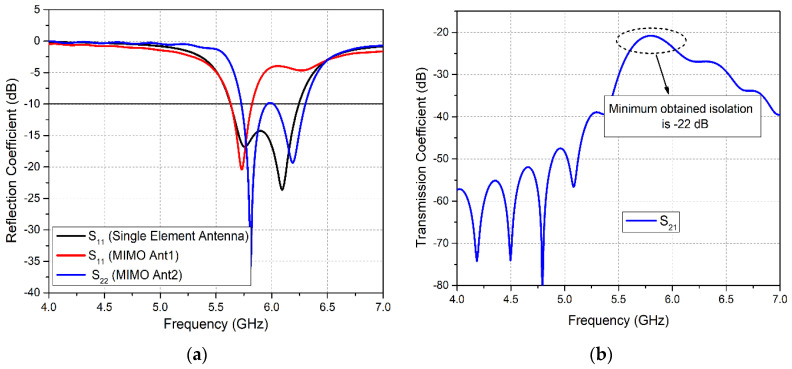
Simulated S-parameter Curves for LTE Antenna: (**a**) Reflection Coefficient Curves; (**b**) Transmission Coefficient Curve.

The performance of the LTE single patch antenna as well as the MIMO array is investigated through the reflection coefficient and transmission coefficient curves, as shown in Figure 3a,b, respectively. It is clear from the *S*_11_ curve that the single-element LTE antenna is covering the desired LTE-band (47) and part of LTE-band 46, ranging from 5.62 to 6.25 GHz with a sufficient operating bandwidth of 630 MHz. Also, the *S*_11_ and *S*_22_ plots demonstrate that both MIMO elements Ant1 and Ant2 are resonating for the frequency bands 5.63–5.85 GHz and 5.72–5.97 GHz, respectively. A slight band shift is observed for the two elements. In addition, analysis of the S-parameter curves demonstrates that the obtained bandwidth of the operating band for the MIMO configuration has been reduced to 250 MHz as compared to the single-element antenna, due to coupling between the two elements. Furthermore, the transmission coefficient curves in Figure 3b elucidate that a minimum isolation of 22 dB is achieved amid the two MIMO antennas for the entire operating band.

### 2.2. 5G Millimeter-Wave Antenna

**Figure 4:**
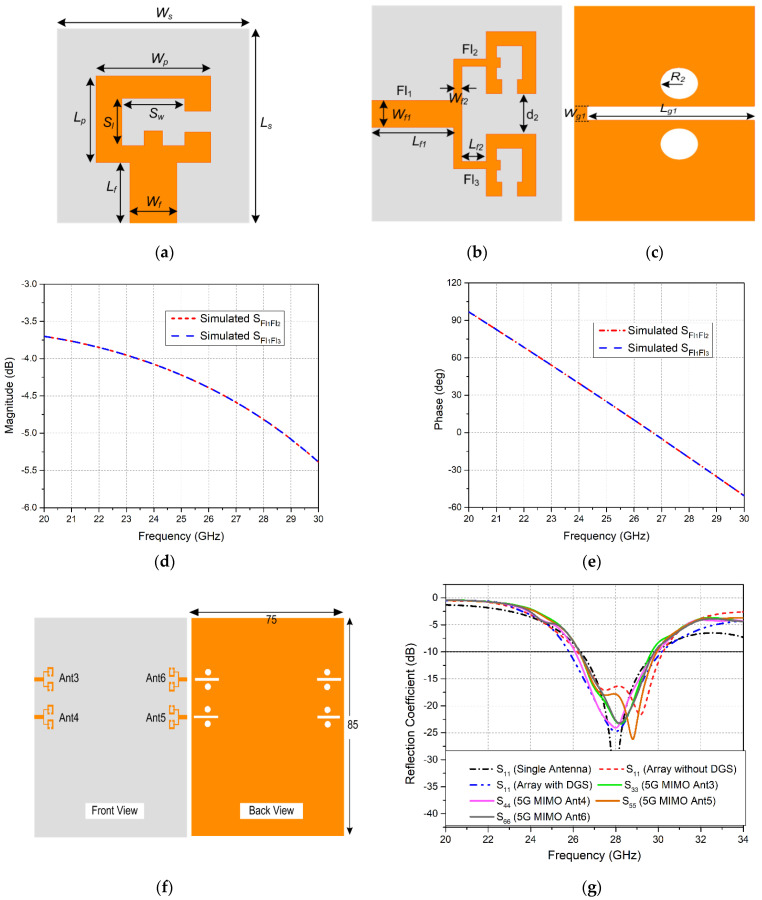
Geometry of 5G antennas: (**a**) Single Patch Antenna; (**b**) 5G Antenna Array, Front View; (**c**) 5G Antenna Array, Back View; (**d**) Simulated Transmission coefficient magnitude curves of the feed network; (**e**) Simulated Transmission coefficient phase curves of the feed network; (**f**) 5G MIMO Design; (**g**) Simulated Reflection Coefficient Curves for 5G Antenna.

Afterward, the design process proceeds to the modeling of the mm-wave 5G antenna. Initially, the primary single-element antenna resonance at 28 GHz is obtained using Equations (1)–(4). The final, optimized 5G antenna is a C-shaped patch, developed by subtracting a rectangular slot of dimensions *sl* × *sw* from the centre of the patch as shown in Figure 4a.

At mm-wave frequencies, it is required that the gain of the antenna should be high to deal with elevated atmospheric attenuation and absorption. However, the single-element antenna obtained in this work does not provide sufficient gain. Therefore, the design was further evolved into a two-element array with a corporate feed network, as exhibited in Figure 4b. The elements of the array are linked by a T-junction shaped parallel feed network, where the feed line widths of the network are considered to match at 50Ω impedance with the main feed, but with the branched network at 100Ω. For impedance matching during modeling, the following transmission line characteristic equations are considered [41].

For $\frac{W_{f}}{H}\leq 1$,

(5) $$Z_{o}=\frac{60}{\varepsilon_{reff}}ln\left[\frac{8H}{W_{f}}+\frac{W_{f}}{4H}\right]$$

where

(6) $$\varepsilon_{reff}=\frac{\varepsilon_{r}+1}{2}+\frac{\varepsilon_{r}-1}{2}\left(\frac{1}{\sqrt{1+12\frac{H}{W_{f}}}}+0.04{\left(1-\frac{W_{f}}{H}\right)}^{2}\right)$$

For $\frac{W_{f}}{H}\geq 1$,

(7) $$Z_{o}=\frac{120\pi \sqrt{\varepsilon_{reff}}}{\frac{W_{f}}{H}+1.393+0.667ln\left(\frac{W_{f}}{H}+1.444\right)}$$

where

(8) $$\varepsilon_{reff}=\frac{\varepsilon_{r}+1}{2}+\frac{\varepsilon_{r}-1}{2}\left(\begin{array}{c} \frac{1}{\sqrt{1+12\frac{H}{W_{f}}}}\\ +0.04{\left(1-12\frac{H}{W_{f}}\right)}^{\frac{-1}{2}} \end{array}\right)$$

where *W_f_* is the width of the feed-line, and *Z*_0_ is the characteristic impedance of the transmission line. For calculation of the width and length (*L_f_*) of the feed network, Equations (9)–(11) are used.

(9) $$W_{f}=\frac{2h}{\pi }\left(B-1-ln\left(2B-1\right)+\frac{\varepsilon_{r}-1}{2\varepsilon_{r}}\left[ln\left(B-1\right)+0.39-\frac{0.61}{\varepsilon_{r}}\right]\right)$$

(10) $$L_{f}=\frac{\lambda }{4\sqrt{\varepsilon_{reff}}}$$

(11) $$B=\frac{60\pi^{2}}{Z_{o}\sqrt{\varepsilon_{r}}}$$

where *B* is a constant used in the inverse design formula, expressed in Equation (9) for a microstrip line of a given characteristic impedance. The feed network is optimized and simulated alone, to give equal magnitude and a similar phase. The transmission coefficient curves for the magnitude and phase, between the input of the 5G array (*Fl_1_*) and the excitation ports of the array (*Fl_2_* and *Fl_3_*) are illustrated in Figure 4d,e, respectively.

The two elements of the array are separated by a distance *d*_2_ = 2.4 mm which is approximately equal to 0.25λ at 28 GHz. Furthermore, Figure 4c demonstrates that the bottom ground of the antenna array is defected with a rectangular slot and two circular slots to reduce the coupling effects amid the two patches of the array. Consequently, the radiation performance of the array has been improved. Afterwards, the 5G antenna array was modified to MIMO configuration by placing two array elements on both elongated sides of the substrate facing each other, as demonstrated in Figure 4f.

The reflection coefficient results of the 5G antenna in Figure 4g show that the single patch antenna covered the mm-wave band ranging from 26.3–30 GHz, thus attaining a wide bandwidth of 3.7 GHz. Moreover, the *S*_11_ curve for the two-element mm-wave array structure without DGS exhibits a band coverage ranging from 26.19–30.25 GHz. Likewise, it is observed that the array structure with DGS acquires a wider band ranging from 25.7–30.2 GHz. Therefore, it is clear from these curves that the 5G antenna array has obtained a bandwidth of 4.06 GHz and 4.5 GHz, respectively without DGS and with DGS configuration. Similarly, reflection coefficient plots for the 5G MIMO antennas in Figure 4g exhibit minimal band shifting with insignificant narrowing of the obtained band. The bandwidth thus obtained is 3.8 GHz, which is sufficient for 5G mm-wave applications.

### 2.3. Integrated Design

Subsequently, the LTE and 5G MIMO antennas are incorporated on the same substrate with dimensions of 75 mm × 110 mm × 0.76 mm, suitable for handheld devices. Primarily, the LTE MIMO antenna module is assimilated at the top edge of the substrate, as demonstrated earlier in Figure 1a. In contrast, the 5G MIMO antennas are loaded on each long edge of the board facing each other. Figure 1b depicts the defected ground layer of the final integrated proposed prototype with rectangular and circular slots of optimized dimensions. Hence, the antenna placement strategy, and compactness of the structure, demonstrates the aptness of the design for forthcoming handheld devices [42].

**Figure 5:**
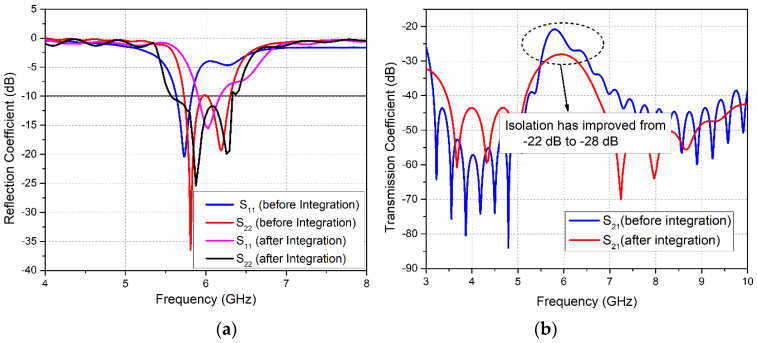
Simulated S-parameter Curves for LTE MIMO Antennas Before and After Integration: (**a**) Reflection Coefficient; (**b**) Transmission Coefficient.

The performance of both MIMO antenna modules was investigated after integration. The reflection coefficient plots of LTE MIMO antennas in Figure 5a demonstrates that before integration, both Ant1 and Ant2 covered the LTE-band 47. After integration, a shift in obtained bands is observed. Ant1 resonates for the frequency range 5.87–6.15 GHz, covering LTE-band 47, while Ant2 now operates at the 5.576.35 GHz band, covering the entire LTE-band 47 and partially covering LTE-band 46. Figure 5a exhibits that, along with the band shift, the bandwidth of the LTE MIMO antennas has also increased after integration. The attained bandwidth for the two antennas was 360 MHz and 780 MHz, respectively. Similarly, the transmission coefficient is another important parameter for the performance analysis of the MIMO antennas. The simulated transmission coefficient curves in Figure 5b depict an improvement in isolation between Ant1 and Ant2 from 22 dB to 28 dB.

**Figure 6:**
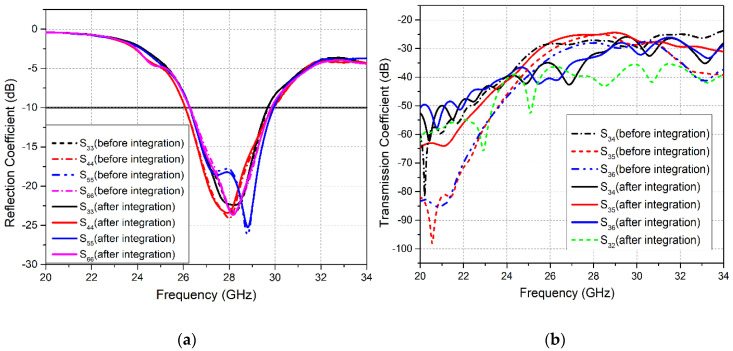
Simulated S-parameter Curves for 5G MIMO Antennas Before and After Integration (**a**) Reflection Coefficient; (**b**) Transmission Coefficient.

In the same manner, reflection coefficient plots for the mm-wave 5G MIMO system in Figure 6a show that after integration all four elements (Ant3–Ant6) are able to resonate at the targeted 28 GHz frequency. The bandwidths obtained for the four MIMO antennas are 3.5, 3.9, 3.7 and 3.6 GHz, respectively. Investigation of the reflection coefficient curves at the mm-wave frequency band demonstrated that integration had caused an insignificant bandwidth reduction from 4.5 GHz to 3.9 GHz. Moreover, investigating the transmission coefficient curves for 5G MIMO antennas in Figure 6b, it is notable that the isolation between Ant3 and Ant5 demonstrated a minimum value of 25.5 dB before integration, and 24 dB after integrating LTE and 5G MIMO antennas on the same board. In contrast, other antennas exhibit better isolation characteristics. However, a slight reduction in isolation is observed between all 5G MIMO antennas.

**Figure 7:**
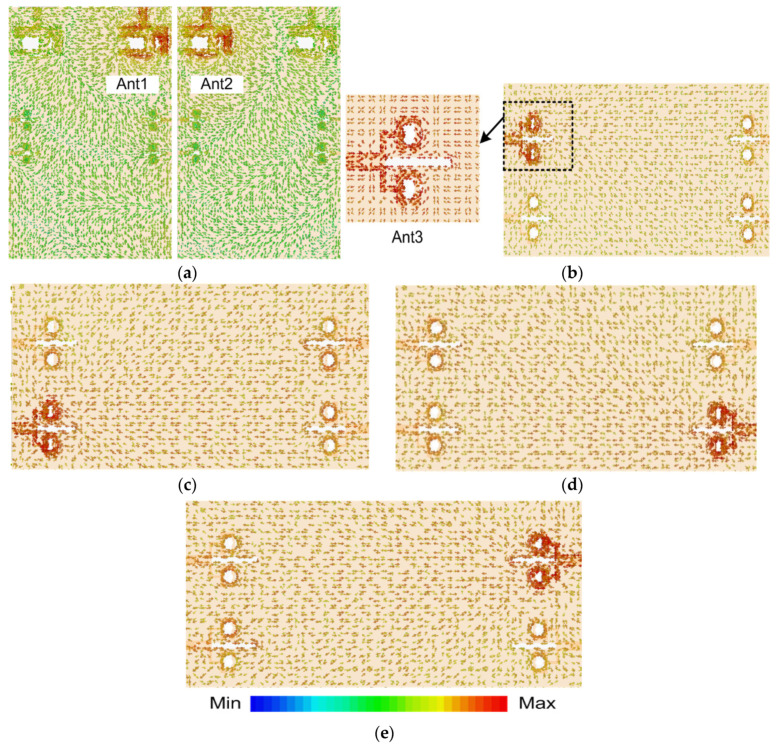
Surface Current Distribution for: (**a**) LTE MIMO Antennas at 5.9 GHz; (**b**) 5G Ant3; (**c**) 5G Ant4; (**d**) 5G Ant5; (**e**) 5G Ant6 (For all 5G Antennas current distribution is investigated at 28 GHz).

In order to substantiate the functioning of the proposed MIMO structure, the simulated surface current densities were examined at the microwave and mm-wave frequencies as shown in Figure 7a–e. This analysis ascertains the radiating antenna parts and also demonstrates the amount of coupling amongst neighboring antenna elements. For LTE MIMO antennas, the analysis was provided at 5.9 GHz. For Ant1, it was observed that the major concentration of current was along the lower strip of the inverted Y-shaped structure as well as on the outer edges of the structure, as depicted in Figure 7a. Likewise, it is illustrated that the current density is widely distributed around the feedline and along the edges of the Ant2. It is also noticeable that some current is generated around the circular slots in the ground plane, which suggests a contribution of defected ground structure to the radiation behavior. Similarly, in order to perceive the radiation and coupling effects between the neighboring elements of mm-wave MIMO configuration, current density was investigated at 28 GHz. Figure 7b illustrates the current distribution when only Ant3 is excited. It was ascertained that surface current is mainly concentrated around the C-shaped slot of Ant3, however some amount of current is also coupled to the adjacent antenna elements. Moreover, surface current was observed along the slots in the ground plane beneath Ant3, affecting the radiation behavior of the antenna. Figure 7c–e demonstrate the current density when Ant4-Ant6 are excited. It is notable that nearly similar distributions exist for all other 5G MIMO antennas. Hence, the distribution paths discussed above have validated the established antenna principles described earlier in this work.

## 3. Experimental Results

**Figure 8:**
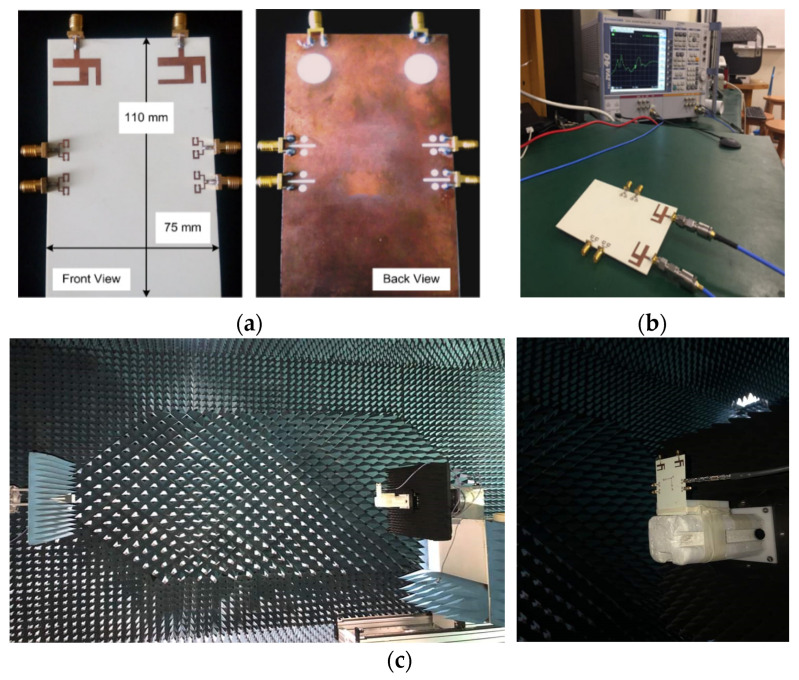
(**a**) Fabricated Prototype, Front and Back View; (**b**) S-parameter measurement with Vector Network Analyzer(VNA); (**c**) Far-field measurement setup.

The proposed integrated MIMO antenna prototype was manufactured on a Rogers RO4350B substrate using the photolithography process, so that the concept could be demonstrated experimentally. Figure 8a–c depict the fabricated prototype, the S-parameters measurement setup, and the setup for the far-field measurements, respectively. The experimental results manifesting the antennas performance are discussed below.

### 3.1. Scattering Parameters

**Figure 9:**
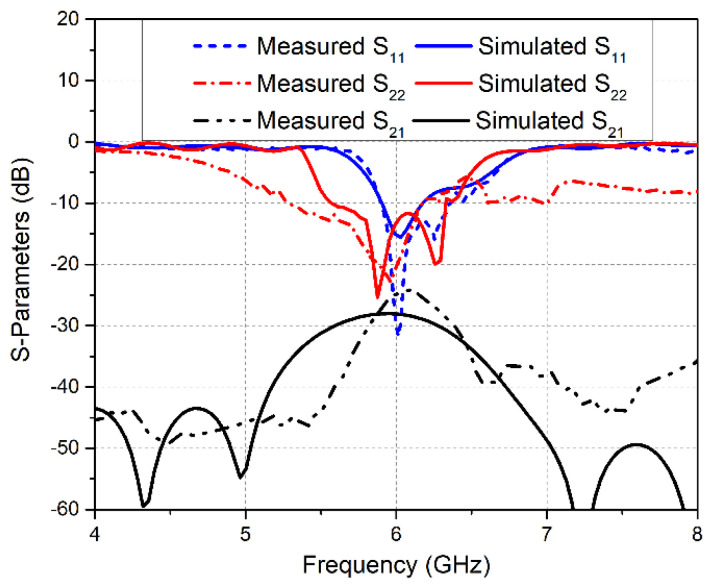
Simulated and Measured S-parameter Curves for LTE MIMO Antennas.

In order to measure the S-parameters of the antenna design, the Rohde & Schwarz ZVA 40 Vector Network Analyzer was used as shown in Figure 8b. The measured reflection coefficient curve S11 for Ant1 (Figure 9) shows that the obtained band ranges from 5.88–6.35 GHz. A widening of the band was observed for the measured result as compared to the simulated S11 plot. Similarly, Ant2 resonates for the 5.29–6.12 GHz frequency band, as illustrated in S22 plot in Figure 9. Comparative analysis of simulated and measured S22 plots demonstrates a band shift. Hence, the measured obtained bandwidths for the LTE MIMO Ant1 and Ant2 were 470 MHz and 830 MHz, respectively. In addition, measured and simulated transmission coefficient plot S21 depicts a decrease in isolation between MIMO Ant1 and Ant2, with a minimum measured isolation of 25 dB.

**Figure 10:**
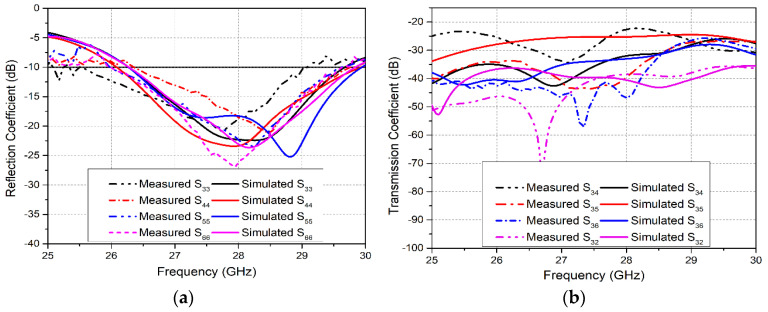
Simulated and Measured S-parameter plots for 5G MIMO Antennas: (**a**) Reflection Coefficient Curves; (**b**) Transmission Coefficient Curves.

In the same manner, measured and simulated S-parameter results for the mm-wave 5G MIMO antennas are shown in Figure 10a,b. The analysis of simulated and measured reflection coefficient plots (*S*_33_, *S*_44_, *S*_55_, and *S*_66_) for Ant3–Ant6 shows band shifts. However, the 5G MIMO antenna system acquires a sufficient measured bandwidth of 3.5 GHz, covering the potential 26/28 GHz mm-wave frequency band, as illustrated in Figure 10a. Furthermore, the measured transmission coefficient plots for the 5G MIMO antennas in Figure 10b demonstrate a minimum isolation of 22 dB between Ant3 and Ant4, as evident from the *S*_34_ curve. Hence, good coherence is realized between the simulated and measured results. However, minor inconsistencies occur due to errors in fabrication as well as the unavoidable use of coaxial cables during the measurement of the antenna [43,44].

### 3.2. Far-Field Results

**Figure 11:**
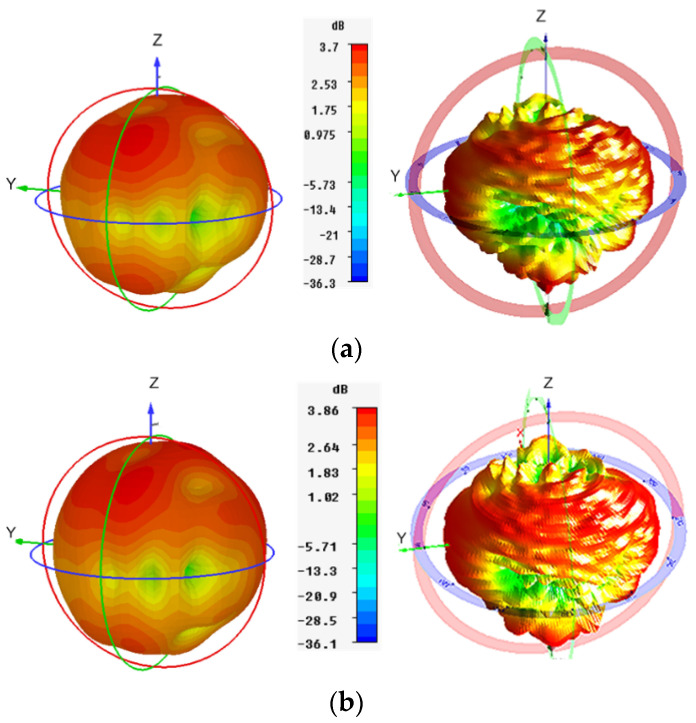
Simulated (left) and Measured (right) 3D Radiation patterns at 5.9 GHz for (**a**) Ant1; (**b**) Ant2.

The 3-dimensional (3-D) radiation patterns of the proposed LTE MIMO antennas were obtained through the commercial ORBIT/FR far-field measurement system in a shielded RF anechoic chamber, as depicted in Figure 8c. The simulated and measured radiation patterns for Ant1 and Ant2 were obtained at 5.9 GHz, as illustrated in Figure 11a,b respectively. It was observed that the maxima of the Ant1 is tilted with respect to Ant2, demonstrating reduced field correlation, which is required for MIMO operation.

**Figure 12:**
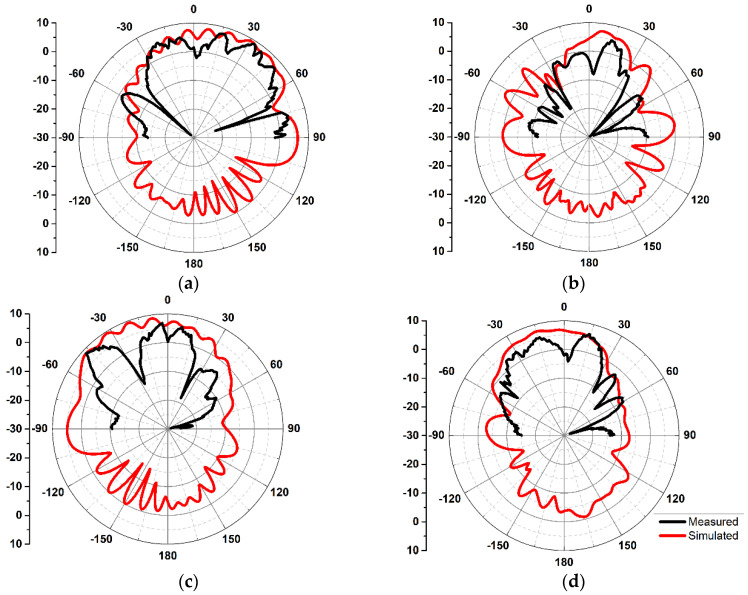
Simulated and Measured 2D Radiation patterns at 28 GHz for: (**a**) 5G Ant3 in the XZ plane; (**b**) 5G Ant3 in the YZ plane; (**c**) 5G Ant6 in the XZ plane; (**d**) 5G Ant6 in the YZ plane.

The 2D radiation patterns for the mm-wave MIMO antennas are depicted in Figure 12a–d. The horn antenna utilized for transmission was an SGH-series horn (SGH-15) by Millitech Co., with 24 dBi standard gain. The far-filed patterns are measured at 28 GHz for theta values in the range from −90° to 90°. The radiation patterns for 5G Ant3 in xz and yz plane are illustrated in Figure 12a,b respectively. The beam maximum was detected at +32° for Ant3 in xz plane. Similarly, 2D radiation patterns for Ant6 exhibit maximum radiation at −45°, as depicted in Figure 12c. The obtained far-field results for the proposed MIMO antennas demonstrate different radiation patterns, which is desirable for MIMO applications.

### 3.3. Gain and Efficiency

The simulated and measured gain as well as radiation efficiency of the demonstrated MIMO configuration at microwave and mm-wave resonating frequencies are summarized in Table 2. The measured maximum gains for LTE Ant1 and Ant2 at 5.9 GHz were 4.86 and 5.13 dB respectively. Similarly, for 5G Ant3 a peak gain of 9.53 dB was attained at 28 GHz, whereas for Ant6 the attained peak gain was 9.31 dB. Moreover, a peak efficiency of 83% was obtained at the lower frequency for the LTE MIMO antennas, whereas for 5G antennas the realized peak radiation efficiency was 73%. The inconsistencies in the simulated and measured results are mainly due to the scattering effect of the connectors and measuring cables.

## 4. Human Safety Concerns (Specific Absorption Rate and Spatial Power Density)

**Figure 13:**
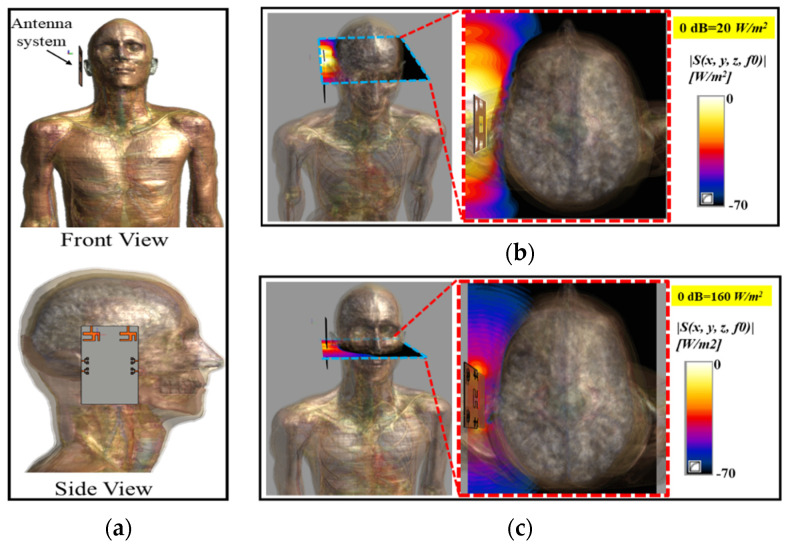
(**a**) Numerical Setup of Antenna, Front and Side View; (**b**) Spatial Power Density at 5.9 GHz; (**c**) Spatial Power Density at 28 GHz.

Mobile devices work in close proximity to the human body; their antenna systems radiate and receive high-frequency electromagnetic waves (EM). Excessive absorption of EM radiation by human tissue is harmful, and produces ionization and heating effects. To guarantee human safety, excessive exposure of human tissues to EM radiations is standardizedby the International Commission on Non-Ionizing Radiation Protection (ICNIRP), the Institute of Electrical and Electronics Engineers (IEEE), and the Federal Communication Commission (FCC). According to IEEE, FCC, and ICNIRP regulations, at lower frequencies (up to 3 GHz, 6 GHz, and 10 GHz, respectively), human exposure is evaluated in terms of Specific Absorption Rate (SAR); however, higher frequency EM waves (above 3 GHz) have very short wavelengths and cannot penetrate deep into human tissues. The higher frequency EM waves are concentrated at the skin and can hardly breach the skin and reach the deep tissues. Hence, at higher frequencies, human safety is evaluated in terms of Spatial Power Density (SPD) instead of SAR [45]. The human exposure in terms of spatial power density for above 3 GHz, 10 GHz, and 6 GHz is limited to 10 W/m^2^ by IEEE, ICNIRP, and FCC, respectively, as provided in Table 3. The proposed antennas were analyzed in terms of SAR and SPD at the lower frequency band (5.9 GHz) and SPD only at the mm-Wave frequency band (28 GHz). The proposed antenna system was analyzed for human exposure assessment by mounting the antenna system on the head of a realistic Duke human model, as depicted in Figure 13. The distance between the ear and the antenna system was kept as 3 mm, and each antenna element was given a 1 W input power. Figure 13a shows the front and side views of the numerical setup for the exposure assessment of the proposed antenna system. Figure 13b,c depict spatial power density distributions at 5.9 GHz and 28 GHz, respectively. The peak PD values in Figure 13 were 20 W/m^2^ and 160 W/m^2^ at 5.9 GHz and 28 GHz, respectively.

**Figure 14:**
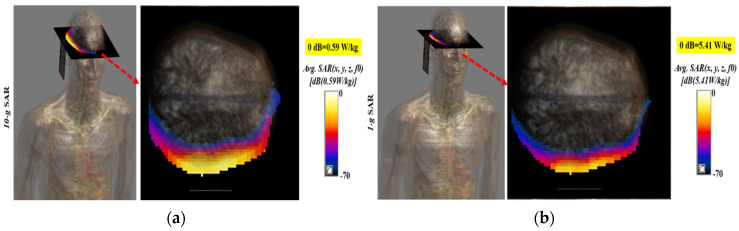
Specific Absorption Rate (SAR) at 5.9 GHz over: (**a**) 1 g; (**b**) 10 g standards.

Figure 14 shows the SAR results for the antenna at 5.9 GHz sampled using 1 g (US standard) and 10 g (European standard) standards. The maximum SAR value of the antenna at 5.9 GHz was 5.41 W/kg for the 1 g standard, while the maximum SAR value of 0.59 W/kg was noted at the same frequency (5.9 GHz) for the 10 g standard, as depicted in Figure 14a,b, respectively. It is worth mentioning here that the peak values were calculated at the input power of 1 W; however, no device, in reality, is allowed to transmit with a power of 1 W. The maximum radiated power for devices operating in the vicinity of the human body is limited to 25 μW [46]. Based on the peak PD values, the maximum allowable powers for the proposed antenna system were calculated as 500 mW and 100 mW at 5.9 GHz and 28 GHz, respectively. This shows that our proposed antenna system is safe, even if it radiated at 500 mW and 100 mW at 5.9 GHz and 28 GHz. The calculated maximum allowable power values are much greater than 25 μW and verify that our proposed antennas system satisfies the human safety standards.

## 5. MIMO Performance Analysis

In order to investigate the MIMO performance of the proposed antenna system, various key metrics such as ECC, DG, MEG, and CCL were investigated. A detailed discussion of the parameters is provided in the subsequent sections.

### 5.1. Envelope Correlation Coefficient (ECC)

**Figure 15:**
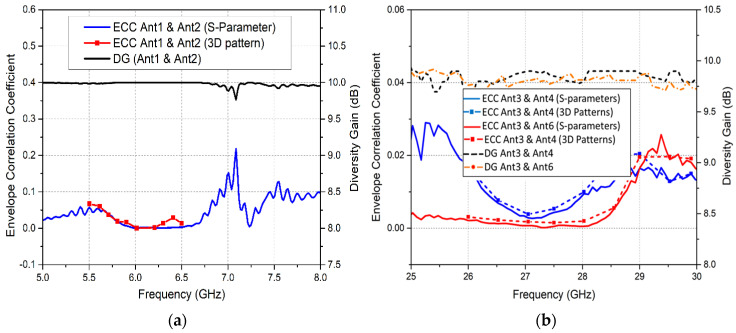
ECC and Diversity Gain for (**a**) LTE MIMO Antennas; (**b**) 5G MIMO Antennas.

ECC is an essential performance parameter for the MIMO antenna, which demonstrates how much MIMO elements are independent from each other. The ECC values for the proposed MIMO antenna system are calculated using the S-parameters or far-field radiation pattern Equations (12) and (13), respectively given in [15,47].

(12) $$\rho_{eij}=\frac{{\left|S_{ii}{}^{*}S_{ij}+S_{ji}{}^{*}S_{jj}\right|}^{2}}{\left(1-{\left|S_{ii}\right|}^{2}-{S_{ij}}^{2}\right)\left(1-{\left|S_{ji}\right|}^{2}-{S_{jj}}^{2}\right)}$$

(13) $$\rho_{eij}=\frac{{\left|\int_{0}^{4\pi }\left[\vec{F_{1}}\;\left(\theta ,\varphi \right)\times \vec{F_{2\;}}\;\left(\theta ,\varphi \right)\right]dΩ\right|}^{2}}{\int_{0}^{4\pi }{\left|\vec{F_{1}}\;\left(\theta ,\varphi \right)\right|}^{2}dΩ\;\int \int_{0}^{4\pi }{\left|\vec{F_{2\;}}\;\left(\theta ,\varphi \right)\right|}^{2}dΩ}$$

where Fi (θ, φ) describes the 3D radiation pattern when the *ith* antenna is excited and the solid angle is represented as Ω in Equation (13). Figure 15a shows the calculated ECC plots, using both S-parameters and 3D radiation patterns for MIMO Ant1 and Ant2, whereas Figure 15b illustrates ECC curves for the mm-wave 5G MIMO antennas. A correlation coefficient value of 0.3 has been set as an acceptable value for wireless systems, with an upper limit of 0.5. The correlation values obtained for the proposed LTE and 5G MIMO antennas were observed to be significantly smaller than the practically acceptable value of 0.5.

### 5.2. Diversity Gain (DG)

Diversity is usually obtained when the transmitter receives multiple path signals. The uncorrelated signals offer higher SNR levels with improved signal reception. Diversity gain describes the amount of reduction in the transmission power without a performance loss after a diversity scheme is used for the MIMO antennas. Diversity gain is obtained using Equation (14) [48].

(14) $$DG=10\sqrt{1-{\left|\rho_{eij}\right|}^{2}}$$

Figure 15a,b exhibit the diversity gain for the LTE and 5G MIMO antennas, respectively. It is notable that a diversity gain of nearly 9.95 dB was obtained for the LTE antennas, whereas for the 5G antennas, a diversity gain of 9.87 dB was obtained for adjacent antennas Ant3 and Ant4. Likewise, for across-the-board antennas Ant3 and Ant6, a diversity gain of approximately 9.83 dB was attained. Therefore, adequate diversity performance is attained for the presented integrated antenna system.

### 5.3. Mean Effective Gain (MEG)

Another important parameter to measure the diversity performance in MIMO system is mean effective gain, which is a ratio of the mean received power to the mean incident power of the antenna. The practically acceptable value of MEG should be

−3 ≤ MEG < −12,

The numerical obtained values of MEG are provided in Table 4. The MEG values for the proposed MIMO antennas validate the above criterion.

### 5.4. Channel Capacity Loss (CCL)

The correlation between the MIMO channel links lowers the MIMO capacity. The capacity loss for MIMO antennas is provided as in [31]:

(15) $$C\left(loss\right)=-log_{2}\det\left(a\right)$$

where a is the 2 × 2 correlation matrix:

(16) $$a=\left[\begin{array}{cc} \sigma_{11} & \sigma_{12}\\ \sigma_{21} & \sigma_{22} \end{array}\right]$$

where

(17) $$\sigma_{\mathrm{ii}}=1-\left({\left|S_{\mathrm{ii}}\right|}^{2}-{\left|S_{\mathrm{ij}}\right|}^{2}\right)$$

(18) $$\sigma_{\mathrm{ij}}=-\left(S_{\mathrm{ii}}{}^{*}S_{\mathrm{ij}}+S_{\mathrm{ji}}S_{\mathrm{jj}}{}^{*}\right)$$

**Figure 16:**
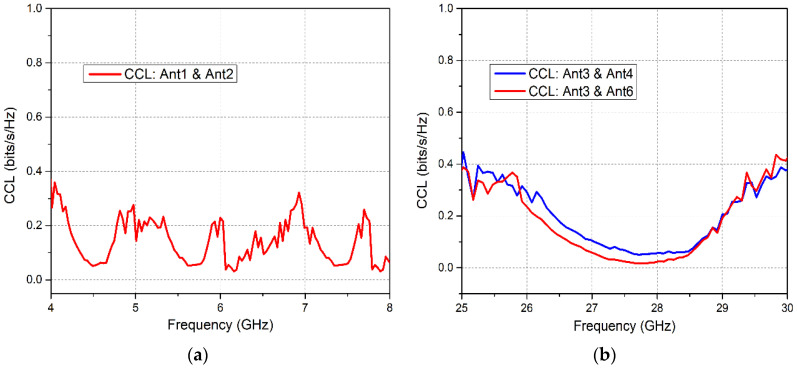
Channel Capacity Loss (CCL) for (**a**) LTE MIMO Antennas; (**b**) 5G MIMO Antennas.

The elements *σii* and *σij* are the correlation coefficients between antennas *i*,*i* and *i*,*j* in an N × N MIMO array. Figure 16a,b demonstrate the channel capacity loss for the proposed LTE and 5G MIMO antennas. It is observed that the obtained CCL was less than 0.4 for the microwave and mm-wave resonant bands, which demonstrates the high data throughput of the proposed system.

## 6. Comparison with Related Works

The proposed integrated 4G/LTE and 5G antenna system is compared with recently published related works to further elucidate the merits of the presented antenna. The comparison, considering a few key design and performance metrics, is provided in Table 5, which demonstrates that the proposed design shows better radiation characteristics in terms of gain, isolation and diversity performance. It is notable that work presented in [33,34], and [37] demonstrates integrated designs. However, mm-wave band is not covered by these works; instead sub-6 GHz 5G bands are covered. In addition, the ECC and gain values are low compared to the proposed design. The work reported in [35,38,39,40] supports a mm-wave 5G band. However, [35] presents a multilayered structure and the antenna footprint is relatively large compared to the proposed work. Additionally, the gain is low in [35]. The work in [38] demonstrates a conformal MIMO integrated antenna solution for 4G and 5G applications. However, the gain attained by the antenna is comparatively low. Similarly, Reference [39] obtains better gain and isolation values for mm-wave 5G antenna compared to the proposed work. However, MIMO configuration is not attained and thus capacity is not enhanced. The antenna footprint, gain efficiency, and isolation of the proposed antenna system is better than the work in [39]. Moreover, only [34] provided the human head/hand phantom analysis. In addition, MIMO performance of the presented design has been analyzed throughseveral parameters such as ECC, DG, MEG, and CCL. The SAR and PD analysis of the proposed design satisfies the international human safety regulations. The obtained results for the proposed work demonstrate that all these parameters are within the practically acceptable range of values. Therefore, the smaller antenna footprint, high gain, good isolation and better diversity performance indicates that the proposed integrated MIMO antenna system is appropriate for future wireless communication devices.

## 7. Conclusions

An integrated 4G/LTE and 5G MIMO antenna system for wireless communication applications is demonstrated in this work. The LTE MIMO antenna system consists of two elements, whereas the 5G module comprises four MIMO elements. The LTE antenna module covered LTE-bands 46 and 47 with a -10 dB maximum measured bandwidth of 830 MHz. Similarly, the measured bandwidth obtained by the mm-wave 5G MIMO antenna configuration was 3.5 GHz, covering the 28 GHz band. Moreover, the design exhibited an isolation of more than 25 and 22 dB at the microwave and mm-wave frequency bands, respectively. In addition, the proposed LTE and 5G MIMO antennas attained peak gains of 5.13 and 9.53 dB, respectively. The calculated values of ECC, DG, MEG, TARC and CCL verified the good diversity performance of the proposed MIMO antennas. Additionally, the proposed antenna system achieved low PD and SAR according to human safety standards. Therefore, the planar footprint, wide bandwidth, high gain and good MIMO performance validates the proposed design as a good contender for integrated 4G/LTE and 5G communication devices.

## Figures and Tables

**Table 1:** Optimized Parametric Values of the Proposed Design.

Name	Value (mm)	Name	Value (mm)	Name	Value (mm)
4G/LTE Antenna parameters					
*L_s_*	25	*W_s_*	21	*L_p_*	11.8
*L_f_*	8.76	*W_f_*	2.2	*W_p_*	19
*L_p_*_1_	3.3	*W_p_*_1_	3.4	*L_p_*_2_	2.5
*W_p_*_2_	3.4	*R*_1_	6		
5G Single-element and Antenna Array Parameters					
*L_s_*	6	*W_s_*	6.2	*L_p_*	4.2
*L_f_*	1.7	*W_f_*	1.5	*W_p_*	3.6
*S_l_*	1.35	*S_w_*	2	*L_f_*_2_	1.28
*L_f_*_1_	4.4	*W_f_*_1_	1.6	*W_f_*_2_	0.4
*L_g_*_1_	12.5	*W_g_*_1_	1.35	*R*_2_	1.3
4G/LTE and 5G Integrated Design					
*L*	110	*W*	75		

**Table 2:** Gain and Radiation Efficiency of Integrated MIMO Antenna System.

Frequency (GHz)	Ant #	Peak Gain (dB)		Radiation Efficiency (%)	
		Simulated	Measured	Simulated	Measured
5.9	Ant1	3.7	5.13	73	71
	Ant2	3.8	4.86	79	83
27.5	Ant3	10.14	9.43	79	71
	Ant6	9.37	9.49	69	72
28	Ant3	9.89	9.53	76	73
	Ant6	9.7	9.31	73	68

**Table 3:** Public Safety Limitations for Electromagnetic Exposure.

Safety Standards	Transition Freq. (GHz)	Power Density Limit (W/m^2^)	Localized SAR Limit below Rransition Frequency (W/kg)
FCC	6	10	1.6 (Averaged over 1 g)
IEEE	10	10	2 (Averaged over 10 g)
ICNIRP	3	10	

**Table 4:** Mean Effective Gain (MEG) for the Integrated Antenna Design.

Frequency (GHz)	MEG (−dB) of Ant No.					
	1	2	3	4	5	6
*5.8*	8.64	6.02	-	-	-	-
*5.9*	6.86	6.03	-	-	-	-
*27*	-	-	6.19	6.44	6.21	7.22
*27.5*	-	-	6.14	6.31	6.12	6.05
*28*	-	-	6.19	6.18	6.06	6.04
*28.5*	-	-	6.37	6.13	6.12	6.10
*29*	-	-	6.92	6.38	6.35	6.30

**Table 5:** Comparison with the Literature Designs.

Figure of Merit	[33]	[34]	[35]	[37]	[38]	[39]	[40]	Proposed
Support 5G (mmWave) band	No	No	Yes	No	Yes	Yes	Yes	Yes
Human safety (SAR/PD) analysis	No	Yes	No	No	No	No	No	Yes
	**4G/LTE Antenna**							
ECC	<0.4	<0.15	0.0058	0.06	<0.04	No	No	≤0.05
Rad. Efficiency (%)	40–60	41–82	75	74	75−90	71, 79	60	71
Gain (dBi)	3.7	Not provided	3.86	3.7	2.15	3.27, 5.41	Not Provided	5.13
MIMO functionality	Yes	Yes	Yes	Yes	Yes	No	No	Yes
	**5G mm-Wave Antenna**							
ECC	<0.2	<0.1	No	No	0.00001	No	No	0.005
Rad. efficiency	62–78	47–79	Not provided	83	Not provided	63	Not provided	73
Gain (dBi)	3.2	Not provided	8	8.5	9	10.29	7	9.53
MIMO functionality	Yes	Yes	No	No	Yes	No	No	Yes
